# Experts' view on the role of oestrogens in combined oral contraceptives: emphasis on oestetrol (E4)

**DOI:** 10.3389/fgwh.2024.1395863

**Published:** 2024-04-09

**Authors:** M. D. Creinin, A. Cagnacci, R. Z. Spaczyński, P. Stute, N. Chabbert-Buffet, T. Korver, T. Simoncini

**Affiliations:** ^1^ Department of Obstetrics and Gynecology, University of California, Davis, Sacramento, CA, United States; ^2^ Academic Unit of Obstetrics and Gynecology, DINOGMI, IRCCS-Azienda Ospedaliera Universitaria San Martino di Genova, Genova, Italy; ^3^ Collegium Medicum, University of Zielona Gora, Zielona Gora, Poland; ^4^ Department of Obstetrics and Gynecology, Bern University Hospital, Bern, Switzerland; ^5^ Gynécologie—Obstétrique et Médecine de la Reproduction—Maternité, Hospital Tenon, Paris, France; ^6^ Reprovision Clinical Consultancy, Oss, Netherlands; ^7^ Division of Obstetrics and Gynecology, Azienda Ospedaliero-Universitaria Pisana, Pisa, Italy

**Keywords:** oestrogens, oestetrol, E4, oral contraception, combined hormonal contraceptives, effectiveness, safety, non-contraceptive benefits

## Abstract

**Introduction:**

The evolution of contraception has been crucial for public health and reproductive well-being. Over the past 60 years, combined oral contraceptives (COCs) have remained an important part of the contraceptive landscape worldwide; continued development has worked toward maintaining efficacy and improving safety.

**Methods:**

Seven global experts convened to discuss the clinical relevance of the oestrogen in COCs, focusing on the impact of the new oestrogen, oestetrol (E4). Participants then commented through an online forum on the summary content and other participants' feedback. We prepared this report to describe the experts' views, their follow-up from the open forum and the evidence supporting their views.

**Results:**

Ethinylestradiol (EE) and oestradiol (E2) affect receptors similarly whereas E4 has differential effects, especially in the liver and breast. Adequate oestrogen doses in COCs ensure regular bleeding and user acceptability. EE and E4 have longer half-lives than E2; accordingly, COCs with EE and E4 offer more predictable bleeding than those with E2. Oestrogen type and progestin influence VTE risk; E2 poses a lower risk than EE; although promising, E4/DRSP VTE risk is lacking population-based data. COCs alleviate menstrual symptoms, impact mental health, cognition, libido, skin, and bone health.

**Conclusion:**

Oestrogens play an important role in the contraceptive efficacy, bleeding patterns, and overall tolerability/safety of COCs. Recent studies exploring E4 combined with DRSP show promising results compared to traditional formulations, but more definitive conclusions await further research.

## 1 Introduction

The development of safe and effective contraception is one of the most significant public health achievements of the 20th century (1). The availability and effective use of modern contraceptives to allow individuals to space or prevent pregnancies results in improved birth outcomes, decreased maternal morbidity and mortality, and improved well-being and population health (2–4). Combined oral contraceptives (COCs) have been available for more than 60 years; many advances have been made during this time, resulting in fewer adverse events while maintaining contraceptive efficacy (5).

In this publication the perspectives of an expert panel on the clinical relevance of the oestrogen component in combined hormonal contraceptives, with a particular emphasis on estetrol (E4), the newest oestrogen introduced for contraception, are presented with the aim of providing better support for oral contraceptive choices.

## 2 Methods

To address the significance of the oestrogen component of combined hormonal contraceptives, including the impact of a new oral contraceptive containing E4, seven international experts (the authors of this paper) were invited to participate in an interactive virtual forum discussion on April 8, 2022. A moderator (MDC) guided a two-hour discussion through open-ended questions covering the following topics:

• The role of oestrogens in combined oral contraceptives.
• Key features of oestrogens in combined oral contraceptives, in relation to contraceptive efficacy and safety.
• The impact of oestrogenicity of COCs on safety.
• The non-contraceptive benefits of COCs, e.g., menstruation symptoms, mood and well-being, sexual life, body weight, skin care, and migraine.
• The effects of oestrogens in COCs used by adolescents and young adults.

The online session was audio-recorded and transcribed, and a summary prepared and posted in an online forum available to the experts. During the 3 weeks following the live session, the experts were asked to comment through the online forum on the summary content and other participants' feedback. In this report, we describe the experts' common views related to the questions, their follow-up from the open forum and the evidence supporting their views.

This publication is intended for healthcare professionals seeking up to date clinical information on the relevance of the estrogenic component in a COC. The main outcomes of the panel discussion related to each question and corroborating references are presented with a summary of the panel members' main points summarized in boxes at the beginning of each section.

## 3 What is the role of oestrogens contained in combined oral contraceptives?

**Table:** 

**Panel discussion summary remarks** • Progestin primarily drives contraceptive effects in COCs by preventing ovulation. • Oestrogen in COCs enhances progestin's effects, stabilizing the endometrium and decreasing the likelihood of irregular bleeding. • COCs with an adequate dose of oestrogen offer consistent, regular bleeding patterns, improving user acceptability. • Reliable bleeding patterns are crucial in contraception, as irregular bleeding often leads to discontinuation. • Comparing trial results can be challenging due to differing definitions for bleeding events and cycle control parameters.

In a COC, the progestin component is primarily responsible for the contraceptive effect, mainly through ovulation inhibition (6, 7). The oestrogen component amplifies the progestational component's effectiveness, enhancing its ability to inhibit gonadotropin secretion and exert antifertility effects within the reproductive tract. The oestrogen also supports endometrial stability, preventing irregular shedding and undesired breakthrough bleeding, resulting in a consistent bleeding pattern (8–11).

The oestrogen-progestin equilibrium required for a regular bleeding pattern is disrupted by using an excessively low dose of ethinylestradiol (EE), as is present in the COC EE 10 µg/norethindrone acetate 1 mg. Substituting EE with 17β-estradiol (E2) or E2 valerate can also result in a less predictable bleeding pattern, likely related to the shorter half-life of E2 in comparison to the progestin (12). In contrast, replacing EE with estetrol (E4) 15 mg in combination with drospirenone (DRSP) 3 mg results most commonly in a consistent and predictable bleeding pattern (11). When estrogen is removed, as with the progestin-only pill (POP) containing DRSP 4 mg in a 24/4 regimen, high rates of unscheduled bleeding and a lack of scheduled bleeding occur as compared to combination products (11).

From a practical, user-orientated perspective, the greatest non-contraceptive advantage of combined oestrogen-progestin pills over progestin-only pills is their ability to produce a consistent, regular bleeding pattern (13). Therefore, a good reason for adding an oestrogen in a contraceptive is to enhance cycle control, users' acceptability, and product continuation (14–17). Nonetheless, comparing results across clinical trials can be challenging due to the varied definitions employed for reporting bleeding events or describing cycle control parameters (18).

## 4 What are key features of oestrogens contained in combined oral contraceptives in relation to contraceptive efficacy and safety?

**Table:** 

**Panel discussion summary remarks** • Oestrogens in COCs act through their effects on oestrogen receptors (ERα and ERβ). • EE and E2/E2 V affect receptor interactions in a comparable manner but at different dose levels. • E4 does not affect ERα receptors universally in the same way as all other oestrogens and is classified as a Native Oestrogen with Selective Action in Tissue (NEST). • The half-life of the oestrogen directly impacts menstrual cycle control. EE and E4, with long half-lives, typically provide a predictable bleeding pattern in combination with a progestin where E2, with a short half-life, commonly results in poor cycle control. • EE increase VTE risk due to liver effects that induce hematologic changes; the impact of EE on the liver is modulated by the progestin component.

The physiological functions of oestrogenic products are modulated by the oestrogen receptors (ER) subtypes alpha (ERα) and beta (ERβ) present in different tissues such as the breast, brain, liver, skin and epithelial and fibromuscular tissues (19). The oestrogens used in COCs all have different properties (20) (see Box 1). These properties relate to the way in which oestrogens exert their effects at their receptors which is more important than their relative amount in the pill (36).

BOX 1 **Key characteristics of oestrogens currently available in COCs.**

**Table:** 

EE • Synthetic oestrogen (21). • ERα selective agonist (22). • Decreases luteinizing hormone to reduce endometrial vascularization. • Diminishes gonadotrophic hormone to prevent ovulation. • Long duration of action (23). • Acceptable oral bioavailability (38%–48%) (21, 24). • Binds significantly to plasma proteins (98.3%–98.5%) (23). • Stronger effect than natural E2 on hepatic metabolism including liver protein synthesis (25). • Half-life (oral) ≈ 24h (26) (≈ 5–30 h) (20). • Increased resistance to metabolism that implies high risk of thromboembolic events (27–29). • The contribution of EE to total estrogenic load in the environment from all sources (including other human pharmaceutical oestrogens, endogenous oestrogens, natural environmental oestrogens, and industrial chemicals), while highly uncertain and variable, appears to be relatively low overall (30). • EE is more persistent in the environment than natural oestrogens and may be a greater cause for environmental concern (31). E2 • Natural oestrogen (20, 32). • ERα and ERß agonist with approximately equal affinity (22) although slightly higher affinity for ERß (20). • Decreases luteinizing hormone to reduce endometrial vascularization (20). • Diminishes gonadotrophic hormone to prevent ovulation (20). • Regulates blood pressure, stimulates endothelial healing and re-endothelialisation, prevents coronary vasospasm caused by endothelial dysfunction, and favours angiogenesis (28, 33). • Low oral bioavailability (2%–10%) (32). • Micronization and esterification needed to enhance oral bioavailability (24, 34). • E2 valerate is the synthetic valerate ester of E2 (35). • Binds significantly to plasma proteins (>95%) (32). • Half-life (oral) ≈ 12 h–20 h (20, 32). • Half-life of E2 in the environment is short and can be biodegraded relatively rapidly (31). E4 • Native, human-specific oestrogen, produced predominately by the foetal liver during pregnancy only (28, 36, 37). • Native Estrogen with Selective Activity in Tissues (NEST) with distinctive vascular and metabolic effects (28, 36). • Activates the nuclear ERα but antagonizes/agonizes (mixed) the membrane ERα. (28). • High oral bioavailability (70%) (28, 38, 39). • High ER specificity diminishes the risk of effects on off-target tissues (36). • Limited impact on the liver: limited impact on the production of liver proteins (36). • Low impact on normal and malignant breast tissue, on haemostasis parameters, coagulation factors, coagulation inhibitors, fibrinolysis, angiotensinogen, triglycerides, and cholesterol, and bone and insulin-like growth parameters (28, 36, 40, 41). • Binds moderately to plasma proteins (<50%); minimal binding to SHBG (36). • Half-life (oral) ≈ 28 h (20, 36). • Predicted environmental exposure to E4 will not affect the aquatic ecosystem (37).

EE, ethinylestradiol; E2, oestradiol; E4, oestetrol; ER, estrogen receptor; SHBG, sex hormone binding globulin.

E4 activates the nuclear ER*α*, but it antagonizes/agonizes (mixed) the membrane ERα, in contrast to other oestrogens (20, 28). Because of this differential mechanism of action, it is classified as a Native Oestrogen with Selective Action in Tissue (NEST). The selective tissue activities of E4 are the consequence of its unique pharmacological profile (42), displaying distinct effects on different tissues which implies a limited impact on the production of liver proteins, and on haemostasis, endocrine and metabolic parameters, rendering a better safety profile compared with other oral oestrogens (28, 36, 39, 40, 42). E4 is a terminal oestrogen that does not convert into other oestrogens and has a long half-life of about 28 h (20, 36, 43). EE also has a prolonged half-life of up to 30 h (20, 44, 45) and therefore both EE and E4, with a progestin, typically offer a predictable bleeding pattern (43, 46). However, EE has been linked to an increased risk of venous thromboembolism (VTE); its dosage and the type of progestin in an EE based COC play crucial roles in modifying this risk (47). Additionally, EE significantly affects the cytochrome P450 system influencing the metabolism of other drugs (23) (see Box 2). Oral E2, with a short half-life of up to 20 h (20), requires micronization to extend its half-life, yet still it tends to result in poor cycle control (32, 45). E2 and E4 in COCs may have a lesser impact on stimulating coagulant proteins compared to EE (20) (Box 1).

BOX 2 **Most important effects related to oestrogens and E4 (36, 48).**

**Table:** 

Target tissue (receptors involved)	Actions of oestrogens	E4 differential characteristics
Adipose tissue (ERα; ERß) (49, 50)	• Adipogenesis • Adipose tissue metabolism	ERα inhibition Prevents weight gain and steatosis (animal finding) Enhances energy expenditure (animal finding) (51)
Bone (ERα; ERß) (49, 50, 52)	• Bone turnover, growth • Osteoporosis prevention	Dose-related decrease of biomarkers of osteocalcin, bone resorption, and bone formation (50, 53)
Brain (ERα; ERß) (49, 54)	• Neuroprotection • Pain sensitivity • Inhibition of inflammation • Memory	ERα activation (49)
Breast (ERα; ERß) (49, 55)	• Tissue (normal and neoplastic) proliferation	Inhibition Antiestrogenic effects on breast cancer cells (55)
Heart (ERα; ERß) (49)	• Cardio-protection	ERα activation (36)
Liver (ERα) (56)	• Cholesterol production • Clotting factors production	Minimal effect Minimal interference with liver cells and drug metabolism (57) Minimal impact on SBHG, coagulation factors, and triglycerides (58)
Ovaries (ERα; ERß) (49, 54, 59)	• Follicle growth	Inhibition Prevents ovulation (59)
Uterus (ERα) (60, 61)	• Endometrial proliferation	ERα activation (61)
Vascular system (ERα; ERß) (28, 49)	• Vasodilation • Atherosclerosis prevention	ERα activation (28, 49)

E4, estetrol; ERα, oestrogen receptor alpha; ERß, oestrogen receptor beta; SBHG, sex hormone binding globulin.

## 5 How can oestrogenicity of COCs impact safety?

**Table:** 

**Panel discussion summary remarks** • Evidence linking COCs to increased VTE risk emerged soon after their introduction. • VTE risk was higher in COCs containing 50 μg or more of oestrogen (mestranol or EE) compared to lower-dosage preparations. • The type of oestrogen in the contraceptive and progestin influences VTE risk. The progestin component modifies this oestrogen effect and has no direct effect on VTE risk. • E2, while less thrombogenic than EE, did show lower VTE risk in a study with E2/nomegestrol acetate (NOMAC) compared to EE/levonorgestrel (LNG) COCs. • E2 V/dienogest (DNG) appears to have a VTE risk similar to EE/LNG-containing COCs. • E4 15 mg combined with DRSP 3 mg shows less pronounced changes in thrombosis factors compared to EE 20 µg/DRSP 3 mg and similar or smaller changes compared to EE 30 µg/LNG 150 µg. This could translate to a low potential for VTE and suggests a risk less than or similar to EE 30 µg /LNG 150 µg. Population-based data are lacking.

Evidence suggesting that oral contraceptives were associated with an increased VTE risk appeared rapidly after they were marketed (62). VTE occurred more frequently with COCs containing more than 50 μg of oestrogen, either mestranol or EE, compared to preparations containing a lower dosage (62). However, oestrogens differ in their effects on thrombotic markers irrespective of the progestin, and newer progestins may affect the impact of oestrogens on haemostatic markers differently than older progestins (62). For instance, the combination of E4 with DRSP resulted in less pronounced alterations in thrombosis factors compared to EE 20 µg/DRSP 3 mg, and comparable or smaller effects compared to EE 30 µg/LNG 150 µg (63). Also, E2 has less of an effect than EE on thrombosis parameters (13, 20). For instance, VTE in E2 COC users has been estimated at 20 cases in 61,600 woman-years (3.2/10,000 users per year) and in EE/LNG COC users at 28 cases in 62,807 woman-years (4.5/10,000 users per year) (64).

Thrombotic marker data are encouraging but are not sufficient to fully assess VTE risk, which requires more research, in particular large scale population based studies (63). Until now, limited epidemiological evidence suggests that E2 V/dienogest (DNG) may have an estimated VTE risk similar to LNG containing COCs (65), or lower vs. EE/LNG (66) while a population-based phase 4 study of E2/nomegestrol acetate (NOMAC) revealed a notably reduced risk of VTE in comparison to EE/LNG COCs [HR: 0.31 (95% CI, 0.13–0.75)] (67). Across the full E4/DRSP clinical program (pooled phase 2 and 3 trials) one VTE case was reported and the estimated annual VTE incidence was relatively low (3.66/10,000 woman-years). This relatively low risk was also reported in a recent modelling study that used the Activated Protein C Resistance as a predictor of VTE risk (68).

Overall, COCs containing natural oestrogens may be preferable over those with synthetic oestrogens due to their lower liver impact. Currently, the clinical and real-world data regarding E2 suggest a VTE profile that may be safer than EE. This promising finding raises expectations that a similar trend may be observed with the non-synthetic oestrogen E4 when combined with DRSP. Nevertheless, definitive conclusions regarding the VTE risk await the completion of post-marketing surveillance studies (40, 63, 69).

## 6 What are the non-contraceptive benefits of COCs?

**Table:** 

**Panel discussion summary remarks** • COCs can alleviate various menstruation-related symptoms, including premenstrual syndrome (PMS), premenstrual dysphoric disorder (PMDD), heavy menstrual bleeding, dysmenorrhea, and irregular bleeding. • Oestrogens in COCs may have positive effects on mental health, mental performance, and cognition; and may also positively influence libido, sexual desire, and vaginal lubrication. • COCs are prescribed for dermatological reasons, including the treatment of hirsutism and acne. • Water retention and weight gain is associated with the use of oestrogens. • E4 when combined with DRSP in a 24/4 regimen may offer several non-contraceptive beneficial effects. However, comprehensive insights into these effects are still premature at this stage.

Oestrogens play a vital role in the non-contraceptive benefits of COCs. Using the right oestrogen-progestin combination can alleviate symptoms and signs in various clinical scenarios, as acknowledged by the expert panel. Examples provided include menstrual-related depressive symptoms, heavy menstrual bleeding (HMB), painful periods, and irregular bleeding (70–74). Certain COCs are approved for treating these conditions (75). For example, E2 V/DNG is indicated for HMB treatment (76) and EE 20 µg/DRSP 3 mg for PMDD treatment (77).

Changes in hormone levels, whether natural or due to contraceptives, can significantly impact emotions (78). COCs with E2 may improve mental health and cognition (79–81), potentially easing depressive symptoms in PMDD (82, 83). COCs can influence libido and vaginal health (84, 85). COCs are also used to treat dermatologic conditions like hirsutism and acne due to their antiandrogenic effects (73, 74, 86–89).

Although trials suggest no direct evidence of COCs causing weight gain, patients still commonly associate their use with this outcome (90). COCs containing DRSP, however, may help prevent weight gain (91). Additionally, COCs could affect migraine frequency, especially menstruation-related migraines, with some formulations showing potential for providing relief (92, 93).

The evidence regarding the non-contraceptive effects of E4 on women's health is still at an early stage. Theoretically, due to its extended half-life and limited impact on the liver, E4 might effectively alleviate physical symptoms associated with PMS (94), such as breast tenderness, headaches, and water retention. Pre-clinical studies suggest that E4 may support vaginal health by promoting epithelial proliferation and lubrication (95, 96). Combining E4 with DRSP also seems to have minimal impact on weight gain (97, 98). There is speculation that contraceptives with natural oestrogens like E4 might be preferred by women experiencing menstrual migraines due to potentially lower cardiovascular risks compared to those containing synthetic oestrogens (92). However, further clinical data is necessary for a more comprehensive understanding of the effects of E4 on users' health, especially those effects that will require follow-up of long-term use. It is noteworthy that in combination with DRSP, E4 has demonstrated high user acceptability and satisfaction, with users expressing a willingness to continue with the contraceptive and reporting a sense of well-being (98).

## 7 What are the bone effects of oestrogens in COCs in adolescents and young adults?

**Table:** 

**Panel discussion summary remarks** • Oestrogens play a critical role in bone development during puberty. • Oestrogen deficiency in adolescence may heighten osteoporosis risk later in life. • E4 may impact bone development differently than EE, potentially offering better prevention against bone loss, although further research is needed for confirmation.

Oestrogens are key regulators of bone turnover in both females and males and play a major role in longitudinal and width growth throughout puberty as well as in the regulation of bone turnover (52). Oestrogen deficiency in adolescence might increase the likelihood of osteoporosis, particularly in individuals with high baseline bone turnover (52, 99–101). Limited data exist regarding the effects of COCs containing E4 on bone tissue. E4, when compared to EE, may have a lesser impact on SHBG and androgens, with a potentially positive effect on bone development in teens (36). Initial findings suggest that E4 might be more effective in preventing bone loss and bone demineralization than other EE (36, 43, 102), but further research is necessary to confirm its efficacy.

In general, COCs are popular among adolescents for birth control, but poor adherence can result in contraceptive failure (103). Choosing the most suitable COCs among adolescents should include counselling on the non-contraceptive benefits of COC. Discussions about improvements in HMB and dysmenorrhea, cycle regularity, possibly acne, hirsutism, and premenstrual symptoms, which are all common amongst adolescents, are important (104). Additionally, information about the COC protective effect against endometrial and ovarian cancer can also be provided (104).

## 8 Conclusions

Oestrogens play an important role in the contraceptive efficacy, bleeding patterns, and overall tolerability/safety of COCs. Beyond contraception, oestrogens in COCs also can alleviate menstrual symptoms, have a positive impact on mood, and potentially affect bone health, and other aspects of well-being. All these properties, alongside the risk of VTE are crucial in tailoring contraceptive choices. For adolescents, discussions around adherence and bone health should also shape COC choices. Recent studies exploring E4 combined with DRSP show promising results compared to traditional formulations. However, population-based outcomes and long-term effects will only come with further research. Continuous research promises deeper insights into the nuanced effects of E4 in COCs, enabling more informed and tailored contraceptive decisions.

## Data Availability

The original contributions presented in the study are included in the article, further inquiries can be directed to the corresponding authors.
